# Clinical Implications of Prenatal Ultrasonographic Detection of Horseshoe Kidney: Association with Postnatal Outcomes

**DOI:** 10.3390/diagnostics16142212

**Published:** 2026-07-15

**Authors:** Suhra Kim, Ju-hee Yoon, Yun Ji Jung, Hayan Kwon, JoonHo Lee, Ja-Young Kwon, Young-Han Kim

**Affiliations:** Department of Obstetrics and Gynecology, Institute of Women’s Medical Life Science, Yonsei University College of Medicine, Yonsei University Health System, Seoul 03722, Republic of Korea; kimsuhra@yuhs.ac (S.K.); jhyoon95@yuhs.ac (J.-h.Y.); ccstty@yuhs.ac (Y.J.J.); whitekwon@yuhs.ac (H.K.); jleemd@yuhs.ac (J.L.);

**Keywords:** horseshoe kidney, congenital fusion of kidneys, prenatal diagnosis, pregnancy, ultrasonography

## Abstract

**Objectives:** Prenatal diagnosis of horseshoe kidney (HSK), a common congenital renal fusion anomaly of the upper urinary tract, remains challenging. Most cases are identified only after birth; however, the clinical significance of prenatal detection and its impact on postnatal outcomes remains unknown. **Methods:** We investigated infants with postnatally confirmed HSK born at a tertiary referral center between January 2009 and August 2025. Prenatal ultrasonographic findings and postnatal outcomes were reviewed. Patients were classified into three groups according to prenatal detection status: missed HSK (group 1), prenatally detected renal abnormalities without recognition of HSK (group 2), and prenatally identified HSK (group 3). Perinatal outcomes, postnatal urological complications, renal function, and associated anomalies were compared. **Results:** Overall, 29 infants were included in this study. Perinatal outcomes, including gestational age at delivery, birth weight, preterm birth, Apgar scores, need for ventilatory support, and neonatal sex, did not differ significantly among the groups. Hydronephrosis and renal scarring were more frequently observed in group 2 than in the other groups. Other urological outcomes, renal function parameters, and mortality rates were comparable between the groups. **Conclusions:** This adds to the limited longitudinal evidence linking the prenatal to postnatal period and evaluating the full spectrum of prenatal detection in HSK, as well as its association with postnatal outcomes. Postnatal outcomes are closely associated with the presence of renal abnormalities. Therefore, prenatal imaging is more appropriate for identifying associated renal abnormalities than for establishing a definitive diagnosis of HSK.

## 1. Introduction

Horseshoe kidney (HSK) is a common congenital renal fusion anomaly of the upper urinary tract, with a reported prevalence of approximately 0.2–0.25% in the general population [1,2]. In most cases, HSK results from the fusion of the two renal units at their lower poles via an isthmus. This abnormal fusion occurs early in embryological development and disrupts the normal ascent and rotation of the kidneys [3]. It typically occurs between the 5th and 9th weeks of gestation as the metanephric blastema encounter one another in the confined space of the fetal pelvis. Under normal developmental conditions, the kidneys migrate from the pelvic cavity to their permanent lumbar position while undergoing a 90-degree internal rotation. In the case of HSK, this cephalad migration is arrested when the central isthmus encounters the inferior mesenteric artery arising from the abdominal aorta. This mechanical obstruction not only traps the fused renal mass at a lower lumbar level but also prevents the completion of normal rotation, leaving the renal pelves oriented anteriorly or ventrally [4,5]. These anatomical alterations lead to the characteristic high ureteral entry and an increased propensity for urinary stasis. These anatomical abnormalities increase the risk of impaired drainage of the collection system. Although many individuals with HSK remain asymptomatic, the condition predisposes individuals to complications, such as vesicoureteral reflux (VUR), pelvic–ureteric junction obstruction (PUJO), ureteral or renal stones, and urinary tract infections [6,7,8,9].

Prenatal identification of HSK typically relies on the detection of lower renal pole fusion and abnormal renal axis rotation via ultrasonography. However, achieving a clear visualization of the central isthmus remains a significant clinical challenge. Beyond the inherent anatomical complexity, several technical and physiological factors contribute to the low prenatal detection rate of HSK. The diagnostic quality of prenatal ultrasonography is heavily dependent on the acoustic window, which can be significantly compromised by maternal factors such as an elevated body mass index or suboptimal amniotic fluid volume. Furthermore, the fetal position plays a critical role; a persistent prone position often causes the fetal spine to create an acoustic shadow that masks the midline isthmus.

The histological composition of the isthmus itself further complicates the diagnosis. While a thick parenchymal isthmus may be visualized as a solid bridge, a thin fibrous isthmus is frequently indistinguishable from the surrounding retroperitoneal tissues on standard two-dimensional imaging. Consequently, even experienced clinicians or sonographers may overlook the fusion if the lateral renal borders appear relatively normal in their respective fossae [10,11]. Due to these multifaceted limitations, the prenatal detection of HSK remains limited [12]. Although specific imaging criteria and diagnostic approaches, such as the measurement of renal pelvic angles, have been proposed to improve detection [13], a substantial proportion of HSK cases remain undetected before birth and are diagnosed only postnatally.

This diagnostic gap raises a critical but poorly understood question regarding whether a missed prenatal diagnosis or the failure to recognize HSK in utero significantly impacts perinatal or long-term postnatal outcomes. Several prior pediatric studies have evaluated the clinical outcomes of HSK diagnosed in childhood [14,15,16]; however, data on cases identified prenatally and followed longitudinally after birth remain limited. Therefore, the aim of this study was to compare perinatal outcomes and long-term prognoses based on the detection of prenatal ultrasonographic diagnosis of HSK.

## 2. Materials and Methods

### 2.1. Study Design and Procedure

This retrospective study was conducted at a tertiary referral center. The medical records of infants with postnatally confirmed HSK born at our institution between January 2009 and August 2025 were reviewed. Clinical data, including prenatal ultrasonographic findings, postnatal management, and clinical outcomes, were extracted from electronic medical records and analyzed.

All prenatal ultrasonographic evaluations were performed by maternal–fetal medicine (MFM) specialists, MFM fellows, and expert sonographers with >10 years of experience in prenatal diagnosis. Scans were obtained using a multiplanar approach with 2–7 MHz transabdominal transducers on Accuvix V20, WS80A, and HERA W10 (Samsung Medison, Seoul, Republic of Korea), Voluson 730 and Voluson E10 (GE Healthcare Ultrasound, Milwaukee, WI, USA), and iU22 (Philips Ultrasound, Bothell, WA, USA) ultrasound systems.

Prenatal ultrasonographic assessment included evaluation of the kidneys in the coronal, axial, and sagittal planes, with assessment of renal position, renal pelvic orientation, lower pole configuration, renal pelvis dilatation, and visualization of the renal isthmus when possible.

Prenatal ultrasonographic findings suggestive of HSK include the presence of an isthmus connecting the lower poles and medial orientation of the lower renal poles, which are characteristic features of this anomaly [13,17]. Postnatal clinical data were subsequently reviewed to confirm the diagnosis and evaluate clinical outcomes. Follow-up duration was defined as the interval from birth to the last available pediatric nephrology, pediatric urology, or imaging follow-up. Long-term renal outcomes were assessed by reviewing renal and bladder ultrasonography, renal function tests, available imaging studies including dimercaptosuccinic acid (DMSA) renal scintigraphy and voiding cystourethrography when performed, urological complications, renal scarring, the need for urological surgery, chronic kidney disease, and mortality. Renal scarring was diagnosed based on DMSA renal scan findings, defined as decreased cortical uptake with contour defects or focal cortical defects. For patients who did not undergo a DMSA renal scan, the absence of documented renal scarring was determined from available follow-up imaging reports and clinical records.

**Figure 1:**
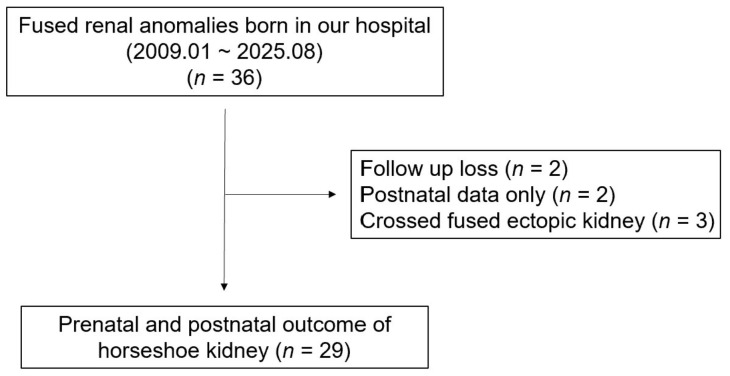
Flow diagram of study population selection. During the study period, 36 infants born at our hospital were diagnosed with renal fusion anomalies. After excluding patients with only postnatal data (*n* = 2), loss to follow-up (*n* = 2), and crossed-fused renal ectopia (*n* = 3), 29 patients with prenatal and postnatal data were included in the final analysis.

During the study period, 36 infants were diagnosed with renal fusion anomalies. Of these, two had only postnatal data available, two were lost on follow-up, and three were diagnosed with crossed-fused renal ectopia. The remaining 29 patients with prenatal and postnatal data were included in the final analysis (Figure 1). Only patients with available prenatal ultrasonographic records, postnatally confirmed HSK, and follow-up data were included in the final analysis.

Prenatal diagnosis of HSK was based on the original prenatal ultrasound report and was considered present when HSK was explicitly diagnosed or strongly suspected. The included patients were classified into three groups according to the detection of prenatal ultrasonographic diagnosis: group 1, in which no prenatal renal abnormalities were detected (missed HSK, *n* = 6); group 2, in which prenatal renal abnormalities were detected but HSK was not recognized (*n* = 10); and group 3, in which HSK was identified or strongly suspected on prenatal ultrasonography (*n* = 13). These predefined groups formed the basis for subsequent statistical comparisons. Prenatal ultrasound reports and stored images of prenatally unrecognized HSK cases (groups 1 and 2, *n* = 16) were retrospectively reviewed to identify the presumed primary factor contributing to non-recognition. Detailed prenatal and postnatal ultrasonographic findings of group 2 patients are provided in Table S1.

### 2.2. Statistical Analysis

Quantitative variables were reported as mean ± standard deviation, and categorical variables were expressed as frequencies and percentages. All statistical analyses were performed using IBM SPSS Statistics (version 31.0; IBM Corp., Armonk, NY, USA). Owing to the non-normal distribution of the data and small sample sizes, nonparametric statistical methods were applied. Differences in continuous variables among the three independent groups were assessed using the Kruskal–Wallis test. Categorical variables were analyzed using the chi-square or Fisher’s exact test, as appropriate. In analyses involving three groups, the Fisher–Freeman–Halton exact test was used for 2 × 3 contingency tables when the assumptions of the chi-square test were not met. A two-sided *p*-value < 0.05 was considered statistically significant.

## 3. Results

**Figure 2:**
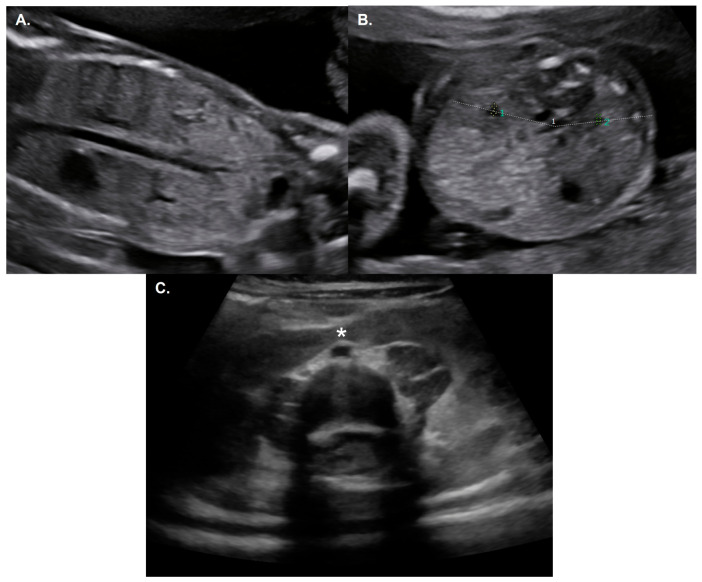
A representative case from Group 1: Postnatal diagnosis of horseshoe kidney (HSK) following unremarkable prenatal ultrasonographic findings. (**A**) Prenatal gray-scale ultrasonography at 20 weeks of gestation demonstrating no apparent renal anomalies in the coronal view. (**B**) Measurement of the renal pelvic angle (160°), a finding highly suggestive of normal renal development during the prenatal period. (**C**) Definitive postnatal diagnosis of HSK confirmed during clinical evaluation in the neonatal intensive care unit. Asterisk (*) indicates the renal isthmus, which was not clearly identified during the prenatal scan. HSK, horseshoe kidney.

**Figure 3:**
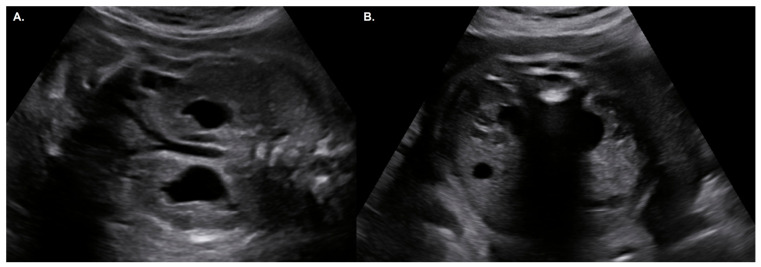
A representative case from Group 2: prenatally detected renal abnormalities without recognition of HSK. Prenatal ultrasonography at 29 weeks of gestation demonstrating bilateral hydronephrosis. While renal pelvis dilatation was identified, the underlying fusion anomaly was not definitively diagnosed during the prenatal period. (**A**) Coronal view and (**B**) Axial view of gray-scale image showing dilated renal pelvis. HSK, horseshoe kidney.

**Figure 4:**
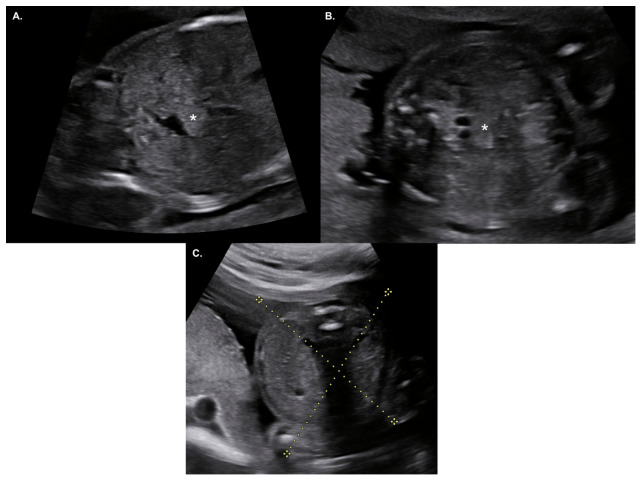
A representative case from Group 3: prenatally detected HSK. (**A**) Prenatal gray-scale ultrasonography in coronal view, demonstrating a horseshoe kidney. The asterisk (*) indicates the renal isthmus connecting the lower poles. (**B**) Prenatal gray-scale ultrasonography in axial view, clearly showing the renal isthmus (*) crossing the midline anterior to the spine. (**C**) Measurement of the renal pelvic angle (80°), which is decreased and highly suggestive of HSK in the prenatal period. HSK, horseshoe kidney.

In analyses comparing the three groups, no significant differences were observed in terms of maternal age, gestational age at diagnosis, and mode of delivery. Neonatal outcomes, including gestational age at delivery, birth weight, preterm birth, Apgar scores, requirement for ventilatory support, and neonatal sex, were comparable across groups (all *p* > 0.05; Table 1). To illustrate the diagnostic challenges and patterns identified in our cohort, representative prenatal ultrasonographic images are presented in Figure 2, Figure 3 and Figure 4. Specifically, Figure 2 highlights the difficulty of detecting HSK when the kidneys appear independent and appropriately positioned in their respective fossae. Figure 3 illustrates a case in which a prominent secondary pathology, such as hydronephrosis, was identified, but the underlying renal fusion anomaly was undetected. Figure 4 shows the definitive identification of the parenchymal isthmus crossing the midline anterior to the great vessels, which allowed for a successful and definitive prenatal diagnosis.

Among the 16 prenatally unrecognized HSK cases, the presumed primary contributing factors were fetal position (*n* = 8, 50.0%), associated renal anomalies masking the underlying fusion anomaly (*n* = 4, 25.0%), advanced gestational age at the time of evaluation (*n* = 3, 18.8%), and maternal obesity with a limited acoustic window (*n* = 1, 6.3%). These findings suggest that non-recognition was mainly related to limited visualization of the renal isthmus or masking by coexisting renal abnormalities.

Regarding urological outcomes, the incidence of hydronephrosis differed significantly among the groups (group 1, 0.0%; group 2, 50.0%; group 3, 7.7%; *p* = 0.034). Renal scarring was observed more frequently in group 2 (30.0%) than in groups 1 and 3, in which no patients exhibited renal scarring (*p* = 0.038). Other urological outcomes, including the incidence of urinary tract infection, VUR, PUJO, ureterocele, ureteral or renal stones, requirement for urological surgery, chronic kidney disease, and mortality, did not differ significantly among the groups (Table 2). Group 2 showed a heterogeneous spectrum of associated renal abnormalities, including hydronephrosis, duplex collecting system, renal hypoplasia, ectopic/dysplastic kidney, and MCDK, as detailed in Table S1. Therefore, the higher frequencies of hydronephrosis and renal scarring in this group should be interpreted with consideration of these coexisting renal pathologies. All deaths (*n* = 3) occurred in group 3; however, the difference was not significant. One death each was attributed to Patau syndrome, pulmonary hypoplasia, and multi-organ failure secondary to heart failure.

Renal function parameters, including serum creatinine levels and estimated glomerular filtration rate at the initial and final follow-ups, did not differ significantly among the groups. Similarly, the prevalence of associated congenital anomalies was comparable among the groups (*p* = 0.422), although the types varied. The spectrum of associated anomalies observed in our cohort is detailed in Table S2. Individual postnatal follow-up duration and key follow-up findings for each patient with HSK are summarized in Table S3.

## 4. Discussion

This study demonstrated that prenatal detection of HSK did not independently determine perinatal outcomes, whereas long-term urological outcomes were more closely associated with the presence of associated renal abnormalities. In our cohort, accurate prenatal diagnosis was not associated with differences in perinatal outcomes, indicating that accurate prenatal identification of HSK alone did not substantially influence the early neonatal clinical course. A plausible explanation is that isolated HSK often represents a relatively benign anatomical variant rather than a condition inherently associated with significant clinical morbidity [13,18].

A noteworthy finding in our study was the significantly higher incidence of postnatal urological complications, particularly renal scarring (30.0%), in Group 2 compared to Groups 1 and 3 (*p* = 0.038). This observation suggests that the clinical prognosis of HSK is mainly determined by coexisting functional abnormalities rather than the anatomical fusion itself. In Group 2, prenatal identification of overt pathologies, such as hydronephrosis, multicystic dysplastic kidney, or duplex collecting systems (as detailed in Table S1), likely created a “diagnostic overshadowing” effect.

The presence of these severe anomalies likely explains why HSK went unrecognized prenatally in this group; the sonographer’s attention was focused on the immediate clinical threat posed by the dilated system or cystic changes. However, these same coexisting conditions are also the primary drivers of long-term morbidity. As highlighted by Taneja et al., children with renal fusion anomalies often remain asymptomatic unless associated with obstructive uropathy or vesicoureteral reflux (VUR) [4]. Our data reinforce this, showing that when prenatal sonography detects a significant renal abnormality, the presence of an underlying HSK should be actively sought. Even if the HSK itself is not the primary diagnosis, these patients represent a high-risk subgroup requiring intensive postnatal surveillance.

Furthermore, our study provides clinical reassurance regarding Group 1, where HSK was prenatally missed. Despite the lack of an in utero diagnosis, these neonates showed perinatal outcomes comparable to those in the prenatally identified group. This suggests that a missed HSK diagnosis, in the absence of other detectable renal or extrarenal markers, does not necessarily lead to adverse neonatal events. This finding aligns with Sagi-Dain et al. (2018), the isolated discovery of HSK prenatally or postnatally often follows a benign clinical course [17]. For clinicians, this implies that failure to detect HSK on routine screening does not negatively impact immediate neonatal health, and prenatal counseling should focus on risk stratification rather than the technical precision of the fusion diagnosis itself.

In contrast to perinatal outcomes, postnatal urological outcomes were more closely associated with concomitant renal abnormalities than with prenatal detection of HSK. In our cohort, hydronephrosis and renal scarring were predominantly observed in group 2. However, group 2 included patients with a broad spectrum of associated renal abnormalities, including hydronephrosis, duplex collecting system, renal hypoplasia, ectopic/dysplastic kidney, and MCDK. Therefore, these findings should not be interpreted as a direct consequence of missed prenatal detection alone, but rather as potentially reflecting the burden of underlying associated renal pathology. The relatively high incidence of renal scarring (30.0%) in this group may be related to chronic urinary stasis or high-grade VUR, often associated with duplicated or dysplastic systems. These conditions may increase the susceptibility to recurrent urinary tract infections, which are known precursors to parenchymal damage. This observation aligns with Li et al. (2024), who reported that surgical success and long-term outcomes in renal fusion anomalies are heavily influenced by the complexity of the pre-existing collecting system anatomy [5]. This observation aligns with previous pediatric studies reporting that urological morbidity in HSK is largely determined by associated renal abnormalities [5,14,15].

HSK is frequently associated with various urological abnormalities. In the present study, the prevalence of urological complications was lower than that reported in most previous pediatric studies. Overall, additional urological abnormalities were identified in 14 of 29 patients (48.3%). Hydronephrosis was observed in 20.6% of patients, a prevalence rate lower than that reported in previous studies (approximately 35–80%) [15,18,19]. The prevalence of VUR, renal scarring, and PUJO was lower in our cohort (3.4%, 12.5%, and 13.8%, respectively), compared with previously reported rates of 19.5%, 24.0%, and 17.1%, respectively [15]. These differences may reflect variations in study populations and diagnostic timing because our cohort included patients identified by prenatal ultrasonography, encompassing asymptomatic or mildly affected patients, whereas many previous pediatric studies primarily evaluated children diagnosed after the onset of clinical symptoms, potentially reflecting more advanced disease at the time of assessment. Furthermore, our long-term follow-up (mean 65 months) allowed us to observe that many infants with prenatally detected HSK remain stable without progressing to overt clinical symptoms if no high-grade obstruction is present at birth. This “lead-time bias” in prenatal screening might explain our lower complication rates compared to postnatal series, where patients often present with complications like urolithiasis or intractable infections. As noted by Taneja et al. (2025), early identification through screening provides a window for preventive management, which may mitigate the severity of long-term urological morbidity [4].

A previous study has reported that renal fusion anomalies are associated with multisystem congenital anomalies, including the VACTERL association (Vertebral anomalies, Anal atresia, Cardiac defects, Tracheoesophageal fistula with or without esophageal atresia, Renal anomalies, and Limb anomalies) [20]. Consistent with these reports, three patients in our cohort were highly suspected to have the VACTERL association.

Beyond these developmental and structural associations, HSK has been reported to occur in conjunction with certain chromosomal and genetic disorders, although syndromic abnormalities are not present in all patients with HSK. Turner syndrome is a well-recognized condition associated with HSK, and some studies have reported that approximately 15–35% of patients with Turner syndrome have concomitant HSK [21,22,23]. In contrast, when HSK is an isolated finding, the likelihood of an associated syndromic disorder is relatively low [17].

In the present study, postnatal karyotype analysis was performed in nine patients, revealing normal karyotypes in all except for one patient with Patau syndrome. In contrast, no syndromic conditions were reported in the study by Taneja et al. [4] Although rare, HSK occurring in conjunction with Patau syndrome and other associated anomalies have been documented in case reports [24], suggesting that chromosomal abnormalities cannot be entirely excluded when additional congenital anomalies are present.

In addition to chromosomal abnormalities, HSK is reportedly associated with rare genetic syndromes, including Fanconi anemia [15] and Kabuki syndrome [25], suggesting that in selected cases, HSK may represent a manifestation of an underlying systemic condition rather than purely an anatomical variant. In our cohort, one case of Fanconi anemia and one case of Kabuki syndrome were identified. Therefore, when HSK is suspected based on prenatal imaging, a systematic and thorough prenatal ultrasonographic evaluation is warranted to identify other associated anomalies.

Based on our case-by-case review of prenatally unrecognized HSK cases (groups 1 and 2, *n* = 16), non-recognition was mainly attributable to fetal position (*n* = 8). These findings suggest that incomplete prenatal detection of HSK may reflect technical and anatomical limitations. Consequently, failure to identify HSK in utero does not necessarily indicate a more severe disease course. This distinction has important implications for prenatal counseling, emphasizing the need to focus on postnatal management strategies and appropriate follow-up rather than solely on prenatal detection.

More importantly, our findings suggest that the clinical implications of prenatal ultrasonography extend beyond confirming the diagnosis of HSK itself. Careful identification of associated renal abnormalities, such as hydronephrosis, duplex collecting system, renal hypoplasia, or dysplastic kidney, may provide more meaningful information for postnatal risk assessment than prenatal recognition of HSK alone. These associated abnormalities were more closely related to subsequent urological complications in our cohort and therefore should be prioritized during prenatal assessment to facilitate appropriate parental counseling, individualized postnatal surveillance, and timely referral for pediatric nephrology or urology evaluation.

The incremental contribution of this study lies not in demonstrating a causal effect of prenatal detection on clinical outcomes, but in describing the clinical spectrum of postnatally confirmed HSK according to prenatal detection status and linking these prenatal patterns with postnatal follow-up findings. Although previous pediatric studies have shown that outcomes in HSK are largely influenced by associated renal and urological abnormalities, data connecting prenatal ultrasonographic detection with long-term postnatal outcomes remain limited. Our findings support the current clinical understanding that prenatal detection of HSK itself does not necessarily determine prognosis, while emphasizing that associated renal abnormalities detected prenatally may be more relevant for counseling, postnatal evaluation, and follow-up planning.

This study has several strengths. First, by including patients accurately diagnosed prenatally and those missed or considered normal during prenatal assessment, it offers a more pragmatic evaluation of the clinical relevance of prenatal detection. Second, the study was conducted at a high-volume tertiary referral center, enabling systematic case accumulation over an extended period, facilitating comprehensive analysis of clinical data. Unlike a previous study with relatively short follow-up durations limited to childhood [15], the present study achieved a long-term longitudinal follow-up with a mean duration of 65.0 months. To our knowledge, this study adds to the limited longitudinal evidence linking prenatal detection of HSK with postnatal outcomes by following patients from the prenatal period through postnatal life and evaluating the full spectrum of prenatal detection status. Third, by demonstrating that postnatal outcomes are more strongly influenced by the presence of associated renal abnormalities than by prenatal detection, this study underscores the clinical importance of risk stratification with direct application to real-world practice. Finally, the integration of this study enhances its clinical applicability by interpreting prenatal ultrasonographic findings in conjunction with postnatal management strategies and follow-up planning. This approach supports prenatal counseling that prioritizes postnatal management over diagnostic precision alone and provides a framework for systematic follow-up of at-risk infants.

This study has some limitations. First, because only postnatally confirmed HSK cases from a single tertiary referral center were included, selection bias may have influenced the findings. Complex or symptomatic cases and those with associated renal or extrarenal anomalies may have been preferentially referred, potentially overestimating the rates of associated anomalies, urological complications, or mortality. Conversely, asymptomatic HSK cases that were not diagnosed after birth or cases delivered at other institutions may not have been captured. Second, the relatively small sample size limits the statistical power of the study, particularly for uncommon outcomes such as mortality, chronic kidney disease, VUR, and other specific urological complications. Accordingly, comparisons involving these rare outcomes should be interpreted as descriptive and exploratory rather than definitive. Third, because of the retrospective design, investigations were performed based on clinical indications; therefore, not all patients underwent uniform, fixed protocols, reflecting real-world clinical practice. Further large-scale multicenter prospective studies are warranted to confirm and generalize these findings.

## 5. Conclusions

This study demonstrated that the prenatal detection of HSK does not directly determine perinatal or long-term outcomes. In contrast, the postnatal urological complications were more frequently observed in patients with associated renal abnormalities than with prenatal detection of HSK itself. These findings support careful prenatal assessment for associated renal abnormalities to improve postnatal risk assessment and follow-up planning, although larger studies are needed for validation.

## Figures and Tables

**Table 1:** Baseline characteristics of infants with horseshoe kidney according to prenatal detection group.

Descriptive Statistics	Group 1 (*n* = 6)	Group 2 (*n* = 10)	Group 3 (*n* = 13)	*p*-Value
**Maternal clinical characteristics**				
Age (y)	35.3 ± 4.2	33.7 ± 3.2	33.2 ± 3.6	0.481
GA at diagnosis (wk)		28.3 ± 4.4	26.0 ± 4.1	0.224
Delivery mode (Cesarean section)	4 (66.7)	8 (80.0)	11 (84.6)	0.719
**Neonatal outcomes**				
GA at delivery (wk)	37.6 ± 3.5	37.4 ± 2.5	36.9 ± 3.2	0.692
Follow-up duration (mo)	93.5 ± 52.5	60.8 ± 50.5	55.1 ± 60.2	0.373
Male	4 (66.7)	6 (60.0)	7 (53.8)	1.000
Preterm birth				
GA < 37 wk	2 (33.3)	4 (40.0)	4 (30.8)	0.884
GA < 34 wk	1 (16.7)	1 (10.0)	2 (15.4)	1.000
Birth weight (g)	2570.0 ± 768.7	2673.0 ± 636.5	2673.1 ± 862.3	0.959
Birthweight < 2500 g	3 (50.0)	5 (50.0)	4 (30.8)	0.579
SGA	2 (33.3)	4 (40.0)	4 (30.8)	0.884
1 min AS < 7	2 (33.3)	4 (40.0)	5 (38.5)	1.000
5 min AS < 7	0 (0.0)	1 (10.0)	2 (15.4)	1.000
Ventilatory support (intubation)	2 (33.3)	2 (20.0)	5 (38.5)	0.599

Data are presented as the mean ± standard deviation or number (percentage). *p*-values were calculated using Fisher’s exact test. AS, Apgar score; GA, gestational age; SGA, small for gestational age; y, years; wk, weeks; mo, months.

**Table 2:** Postnatal and long-term outcomes of renal complications according to prenatal detection group.

Descriptive Statistics	Group 1 (*n* = 6)	Group 2 (*n* = 10)	Group 3 (*n* = 13)	*p*-Value
**Postnatal renal and urologic outcomes**				
UTI	1 (16.7)	3 (30.0)	2 (15.4)	0.837
VUR	0 (0.0)	1 (10.0)	0 (0.0)	0.552
Hydronephrosis	0 (0.0)	5 (50.0)	1 (7.7)	**0.034**
PUJO	0 (0.0)	1 (10.0)	1 (7.7)	1.000
Ureterocele	0 (0.0)	1 (10.0)	0 (0.0)	0.552
Ureteral or renal stone	0 (0.0)	1 (10.0)	1 (7.7)	1.000
Renal scarring	0 (0.0)	3 (30.0)	0 (0.0)	**0.038**
Postnatal urological surgery	1 (16.7)	2 (20.0)	0 (0.0)	0.207
Chronic kidney disease	0 (0.0)	0 (0.0)	1 (7.7)	1.000
Death	0 (0.0)	0 (0.0)	3 (23.1)	0.285
**Renal function outcomes**				
Serum creatinine at initial evaluation (mg/dL)	0.55 ± 0.21 (*n* = 5)	0.46 ± 0.18 (*n* = 10)	0.58 ± 0.29 (*n* = 7)	0.564
eGFR at initial evaluation (mL/min/1.73 m^2^)	88.0 (*n* = 1)	87.8 ± 7.1 (*n* = 3)	90.9 ± 32.4 (*n* = 3)	0.985
Serum creatinine level at final follow-up (mg/dL)	0.41 ± 0.10 (*n* = 6)	0.43 ± 0.14 (*n* = 10)	0.56 ± 0.43 (*n* = 8)	0.468
eGFR at final follow-up (mL/min/1.73 m^2^)	82.58 ± 11.97 (*n* = 4)	84.28 ± 6.17 (*n* = 5)	84.83 ± 2.35 (*n* = 4)	0.912
**Associated anomalies**	4 (66.7)	6 (60.0)	5 (38.5)	0.422

Data are presented as the mean ± standard deviation or number (percentage). *p*-values were calculated using Fisher’s exact test. Significant associations are indicated in bold. UTI, urinary tract infection; PUJO, pelvic–ureteric junction obstruction; VUR, vesicoureteral reflux; eGFR, estimated glomerular filtration rate.

## Data Availability

Data is contained within the article or Supplementary Materials.

## Supplementary Materials

The following supporting information can be downloaded at: https://www.mdpi.com/article/10.3390/diagnostics16142212/s1. Table S1: Prenatal and postnatal ultrasound findings (10 cases of renal abnormalities in group 2); Table S2: Postnatally confirmed associated anomalies by study group; Table S3: Individual postnatal follow-up duration and key follow-up findings in patients with horseshoe kidney.

## Abbreviations

The following abbreviations are used in this manuscript:

HSK	Horseshoe kidney
VUR	Vesicoureteral reflux
PUJO	Pelvic–ureteric junction obstruction
MFM	Maternal–fetal medicine
