# Supplementary material for: Clinical Implications of Prenatal Ultrasonographic Detection of Horseshoe Kidney: Association with Postnatal Outcomes
Source: Diagnostics (Basel). 2026 Jul 15;16(14):2212. doi: 10.3390/diagnostics16142212 (PMC13407429; doi:10.3390/diagnostics16142212)
Supplement: Supplementary file 1 [file diagnostics-16-02212-s001.zip › diagnostics-4309557-supplementary.pdf]

Table S1. Prenatal and postnatal ultrasound findings (10 cases of renal abnormalities in group 2)

Prenatal ultrasound findings	Postnatal ultrasound findings
Rt kidney hypoplasia	HSK
Lt renal duplication and Rt small kidney	HSK with Lt hydronephrosis, grade 2
Lt renal agenesis	HSK deviated to the Rt renal fossa
Lt renal duplication with hydronephrosis of both poles (upper pole grade 4, lower pole grade 2), hydroureter of upper pole, ureterocele	HSK with Lt duplicated collecting system, upper moiety hydroureteronephrosis with ectopic ureter insertion, and mild renal pelvic dilatation in the lower moiety
Lt hydronephrosis, grade 4, suspected UVJ obstruction, hydroureter	HSK with marked hydronephrosis and mild parenchymal thinning in the Lt kidney; suspected UPJ obstruction
Lt ectopic kidney with dysplastic change	HSK with Lt MCDK
Rt pyelectasis with dysplastic change	HSK with Rt hydronephrosis, grade 2
Rt small kidney with pelvis displaced medially	HSK without remarkable finding.
Lt renal duplication	1. HSK with increased cortical echogenicity<br>2. Bilateral extrarenal pelvic dilatation (AP diameter: Rt 4 mm, Lt 9 mm)
Bilateral hydronephrosis with hydroureter (Lt grade 3, Rt grade 2)	HSK with bilateral renal and extrarenal pelvic dilatation

Lt, left; Rt, right; HSK, horseshoe kidney; UPJ, ureteropelvic junction; UVJ, ureterovesical junction; AP, anteroposterior; MCDK, multicystic dysplastic kidney

Table S2. Postnatally confirmed associated anomalies by study group

Group	Associated anomalies (per patient)	*n*
Group 1 (*n* = 4)	Filum terminale lipoma	1
	Left ovarian cyst	1
	VSD	1
	Double-chambered right ventricle	1
Group 2 (*n* = 6)	TEF	2
	Chiari malformation	1
	Imperforate anus	1
	Cleft palate, VSD, ventriculomegaly, hearing impairment, tethered cord syndrome	1
	Persistent patent ductus venosus	1
Group 3 (*n* = 5)	Patau syndrome	1
	Bilateral left superior vena cava	1
	VSD, pulmonary hypoplasia, OHVIRA syndrome, left MCDK, hypertelorism, low-set ear	1
	Polydactyly of the right foot	1
	Complete tracheal ring (congenital tracheal stenosis), ectopic kidney, hemivertebra	1

MCDK, multicystic dysplastic kidney; OHVIRA, obstructed hemivagina and ipsilateral renal anomaly (Herlyn–Werner–Wunderlich syndrome); TEF, tracheoesophageal fistula; VSD, ventricular septal defect
